# Tafel Slope Plot as a Tool to Analyze Electrocatalytic Reactions

**DOI:** 10.1021/acsenergylett.4c00266

**Published:** 2024-04-01

**Authors:** Onno van der Heijden, Sunghak Park, Rafaël E. Vos, Jordy J. J. Eggebeen, Marc T. M. Koper

**Affiliations:** Leiden Institute of Chemistry, Leiden University, 2333 CC Leiden, The Netherlands

## Abstract

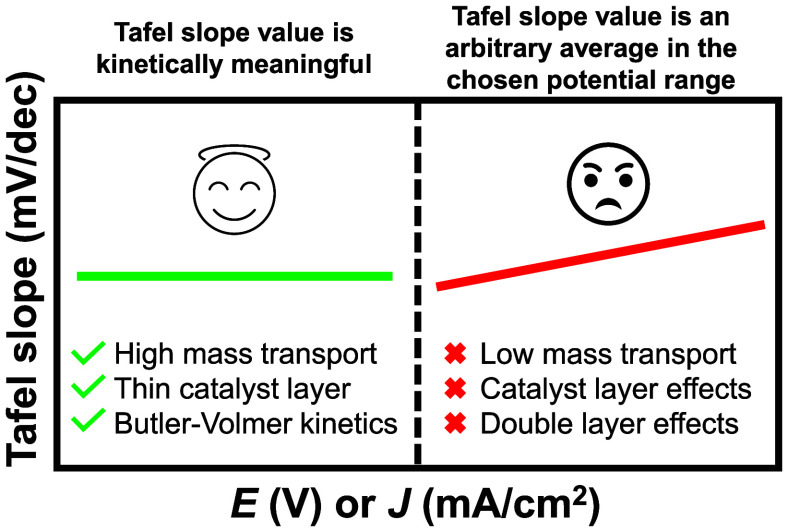

Kinetic and nonkinetic contributions to the Tafel slope value can be separated using a Tafel slope plot, where a constant Tafel slope region indicates kinetic meaningfulness. Here, we compare the Tafel slope values obtained from linear sweep voltammetry to the values obtained from chronoamperometry and impedance spectroscopy, and we apply the Tafel slope plot to various electrocatalytic reactions. We show that similar Tafel slope values are observed from the different techniques under high-mass-transport conditions for the oxygen evolution reaction on NiFeOOH in 0.2 M KOH. However, for the alkaline hydrogen evolution reaction and the CO_2_ reduction reaction, no horizontal Tafel slope regions were observed. In contrast, we obtained the expected Tafel slope of 30 mV/dec for the HER on Pt in 1 M HClO_4_. We argue that widespread application of the Tafel slope plot, or similar numerical differentiation techniques, would result in an improved comparison of kinetic data for many electrocatalytic reactions when the traditional Tafel plot analysis is ambiguous.

Tafel slopes^1^ are a commonly used metric for assessing rates and mechanisms of electrocatalytic reactions. In the simplest definition, it is the number of mVs required to increase the current by a factor of 10, and thus it is reported in mV/dec Therefore, a low Tafel slope value is an indication of an active catalyst, as a smaller overpotential is required to reach a higher current density. Under certain conditions, kinetic information, such as the rate-determining step, can be extracted from the value of the Tafel slope.^2−4^ These conditions are no significant backward reaction, no contribution of (pseudo-) capacitive current, no mass transport limitations, 100% correction of the ohmic resistance, no change in ohmic resistance during the measurement, fixed temperature, a potential independent number of accessible active sites, no potential-dependent changes to the catalyst, and no other processes contributing to the current.^3,5−7^ For multistep mechanisms with changing surface coverage of reaction intermediates, kinetic analysis will become more complicated, and microkinetic analysis should be used to accurately predict the Tafel slopes.^8−11^ From microkinetic analyses, it was shown that the Tafel slope value does not always remain constant at different applied potentials but can change depending on potential. Furthermore, the determination of the (lack of) pH dependence, reaction orders, temperature dependence of the Tafel slope, or the kinetic isotope effect can provide additional evidence to support the rate-determining step derived from the Tafel slope analysis.^10,12,13^

Recently, we have shown that for the oxygen evolution reaction (OER), Tafel slope analysis is often convoluted by nonkinetic effects, such as bubble formation and (internal) OH^–^ gradients, resulting in apparent Tafel slopes with no obvious kinetic meaning.^14^ To illustrate this more clearly, a Tafel slope plot was introduced, where the Tafel slopes were computed over small potential regions and plotted vs the average current or potential. In such a differential Tafel slope analysis,^15^ horizontal regions would indicate a fundamental or “cardinal” Tafel slope value. The corresponding exchange current density from this horizontal Tafel slope region can then also be obtained unambiguously. A similar method was presented to obtain the Butler–Volmer transfer coefficient α,^16,17^ and as “instantaneous” Tafel slope, which was, for example, applied to investigate how the Tafel slope value depends on the number of cycles.^18,19^ Using the Tafel slope plot, it was found that after an initial value at low current density, there was a continuous increase in the Tafel slope value with increasing current density. This increase in Tafel slope value depended on loading, rotation rate, sonication, and reactant concentration, which should not be the case for kinetically meaningful Tafel slopes. In addition to mass transport or other nonkinetic effects, it is important to note that the Tafel slope value can also appear to be potential dependent because of intermediate surface coverages (as evidenced by microkinetic models^9^) or double layer effects (such as the Frumkin correction).

Other reports claim that the use of linear sweep voltammetry (LSV) (or any potentiodynamic technique) may result in incorrect Tafel slope values because different values were obtained for the OER at different scan rates.^5,20,21^ Therefore, it has been argued that only potentiostatic methods, i.e., chronoamperometry and chronopotentiometry (CA and CP), should be used to allow the system to reach steady-state conditions, suggesting that these steady-state conditions are never reached for potentiodynamic techniques, i.e., linear sweep voltammetry and cyclic voltammetry (LSV and CV).^2,20^ Alternatively, electrochemical impedance spectroscopy (EIS) can be used to determine the Tafel slope.^22−24^ However, more research is needed about how Tafel slope values depend on different methods and different conditions.

Here, we compare the Tafel slope values obtained from LSV to the Tafel slope values obtained by potentiostatic methods (CA and EIS) in the horizontal Tafel slope region in the Tafel slope plot. We show that, at a high rotation rate and relatively high reactant concentration, essentially identical Tafel slope values were obtained using different techniques. Moreover, we extended the use of Tafel slope plots to typical reactions for electrochemical energy research: alkaline HER on Pt and CO_2_RR to CO on Au. We showed that these reactions do not exhibit horizontal Tafel slope regions in the Tafel slope plot under the commonly used conditions. By contrast, for HER on a Pt disk RDE as well as for a Pt microelectrode in 1 M HClO_4_, a Tafel slope of 30 mV/dec was observed over a relatively large potential/current range, indicating a kinetically meaningful Tafel slope value. Therefore, we show that the Tafel slope plot can provide insight into the kinetic meaningfulness of the obtained Tafel slope value for multiple electrocatalytic reactions, and its wide application would be beneficial for obtaining fundamental kinetic insights. It also removes the ambiguity that is often observed in determining the Tafel slopes from (slightly) nonlinear Tafel plots.

Complications in Tafel slope analysis can be caused by a multitude of experimental issues. These are, among others, mass transport limitations, bubble formation, problems with determining the ohmic resistance, and changes in the ohmic resistance with applied potential.^25^ In Figure S1, an example of the effect of bubbles on the surface for the impedance measurement is shown, and how it could lead to overcompensation of the LSV and therefore too low Tafel slope values. Furthermore, differences in apparent Tafel slope values were also reported with different loadings.^26^ However, this should not be the case for a fundamental Tafel slope value as it should be independent of the number of active sites. The observed effect of loading could be due to the conductivity within the catalyst layer or pores that can result in local potential drops or to reactant and product mass transport limitations within the catalyst layer^14,26−31^ resulting in apparent Tafel slope values. Moreover, differences in gas bubble coverage can exist, which is important for the accessible surface area during the measurement.^14,32^ Therefore, it is advisable to use thin layers^23^ with relatively high reactant concentrations under high-mass-transport conditions.^33−37^ An overview of the practical considerations for Tafel slope analysis with a Tafel slope plot is given in Figure 1.

**Figure 1:**
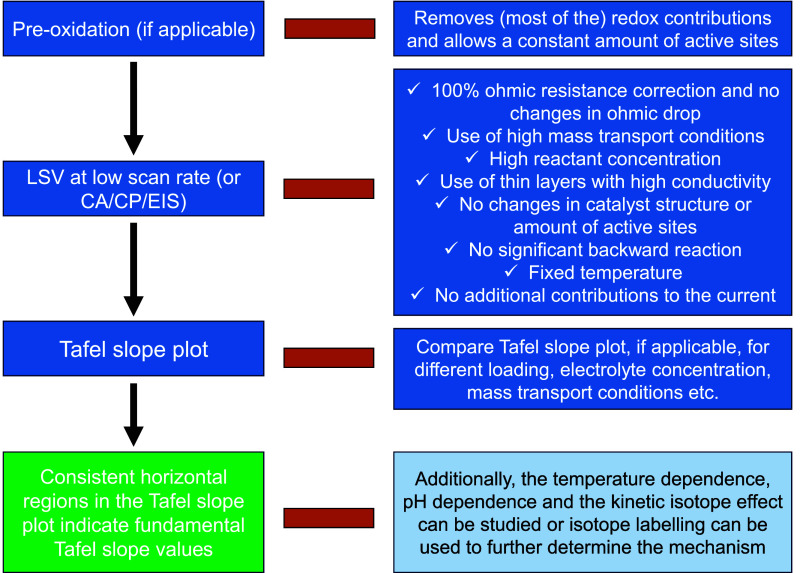
Practical considerations for kinetically meaningful Tafel slope analysis.

To prevent the above-mentioned issues, a high-speed RDE at 4000 rpm with a relatively high concentration of 0.2 M KOH at a relatively low loading of NiFeOOH (generated at 1.4 mA, 5 s; based on a previously reported method;^26^ for more details see the Experimental section) was used to compare three different methods to determine the Tafel slope for the oxygen evolution reaction. Figure 2 shows the Tafel slope plots vs the current (Figure 2A) and potential (Figure 2B). We present a schematic overview for plotting the Tafel slope in Figure S2. In these plots, a horizontal Tafel slope region is observed between 1 and 5 mA/cm^2^_geo_ or 1.465 and 1.490 V. To compare the value obtained from the horizontal region of the Tafel slope plot, we first determined the Tafel slope from the LSV within this horizontal region, showing a Tafel slope of 31.1 ± 0.04 mV/dec (Figure 2C), with the error given being the standard deviation of the fit. In Figure 2D, the Tafel slope was determined from chronoamperometry (CA) data with 1 min stabilization time, and in Figure 2E the Tafel slope was determined from the charge transfer resistance obtained from impedance spectroscopy. These two techniques show Tafel slopes of 30.4 ± 0.3 and 31.7 ± 0.4 mV/dec, respectively, which are very close to the values observed from LSV. Moreover, high *R*^2^ values were obtained for all techniques, although this is not a measure for the correctness of the obtained value. Therefore, under high-mass-transport conditions at a relatively low loading and relatively high reactant (OH^–^) concentration, similar Tafel slope values were obtained in the horizontal region of the Tafel slope plot using both potentiostatic and potentiodynamic techniques. Additionally, if the potential region is extended, the LSV, CA, and EIS measurements all show a similar increasing Tafel slope value, which could indicate an intermediate surface coverage resulting in a potential-dependent Tafel slope value (Figure S3).

**Figure 2:**
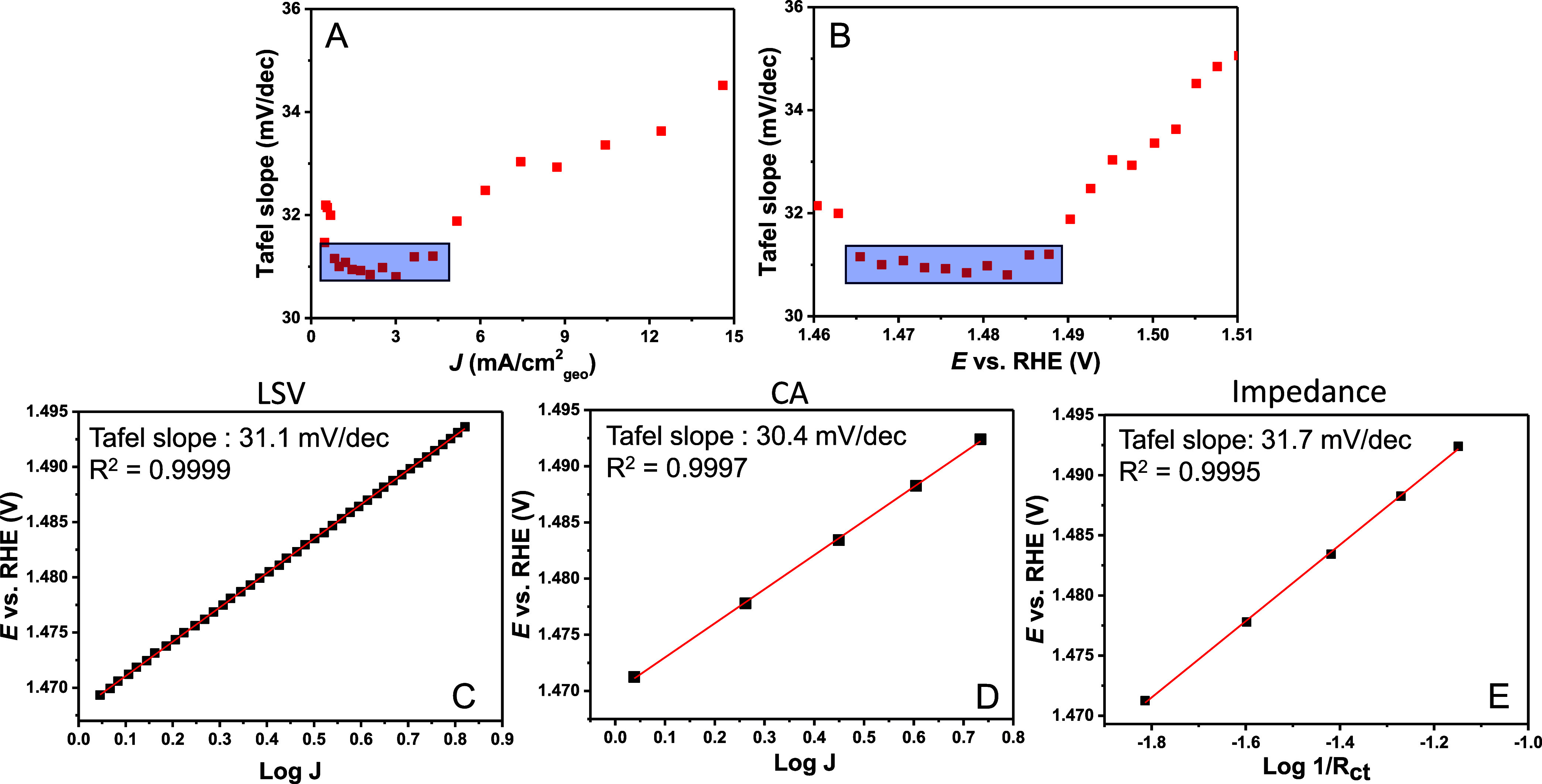
Tafel slope plot of the OER on NiFeOOH in 0.2 M KOH at 4000 rpm with the Tafel slope value calculated over ranges of 10 mV and plotted (A) vs the average current density and (B) vs average potential. (C) Tafel slope determined from the horizontal region in the Tafel slope plot from the linear sweep voltammetry (LSV) at 2 mV/s with 85% *iR* correction during the measurement and 15% manually afterward. (D) Tafel slope from chronoamperometry after 1 min stabilization and 100% *iR* corrected manually afterward (CA). (E) Tafel slope determined from the charge transfer resistance determined by impedance spectroscopy vs the 100% *iR* corrected potential. The Tafel slopes shown in panels C, D, and E were determined within the same potential region. The corresponding LSV, CA, and impedance spectra and data are shown in Figures S3–S7 and Table S1, and the influence of the chosen potential window (5, 10, 15, and 20 mV) to determine the Tafel slope is shown and discussed in Figure S8.

In contrast to the results presented in Figure 2, where no large differences are observed between potentiostatic and potentiodynamic techniques, a strong scan rate dependence has been reported in the literature for the determined Tafel slope of the oxygen evolution reaction.^5,20,21^ This scan rate dependence was explained as an effect of the potentiodynamic nature of the LSV measurement, which would never allow steady-state conditions to be reached; therefore, potentiostatic or galvanostatic measurements were recommended. Figure S9 shows that there is an obvious scan rate dependence on the Ni redox features, but the Tafel slope values become similar at more anodic potentials, as shown in Figure S10. In addition to the redox features, the scan rate also affects the bubble behavior.^14,38^ However, when measured under high-mass-transport conditions (4000 rpm), the LSVs were similar at increasing current density, while at a lower rotation rate (1000 rpm), we observed a scan rate dependence due to gas bubble behavior at higher current density (Figure S9). For CA, CP, or EIS measurements, different numbers of gas bubbles could be dynamically present on the surface, even when the current appears stable, resulting in a different number of active sites at different potentials. Therefore, possible explanations for the reported difference in scan rate is that the scan rate changes the oxidation current contribution most likely at lower overpotentials, and/or the chosen technique influences bubble behavior at higher overpotentials. So while the kinetic current does not depend on scan rate, it would still be advisable to use low scan rates (<5 mV/s), because of (leftover) catalyst oxidation processes at low current densities (Figure S10) or other contributions to the current.

Another potential issue resulting in different Tafel slopes is that oxidation is still required to produce the active site; therefore, a different number of active sites could possibly be found for different measurement potentials. This could be a result of potential drops through pores in large assembled electrodes, as described before,^27−29^ resulting in a slow activation, for example, by the growth of the oxide layer. As a result, the number of active sites becomes a function of the applied potential and/or time. Note that this can affect both potentiostatic and potentiodynamic measurements and depends on the electrochemical procedure. If further oxidation (which may result in additional active sites) still occurs after the onset of the reaction, more active sites at higher overpotentials will result in an apparent Tafel slope that is too low. This can potentially be addressed by preoxidation if the oxidation and reduction features are irreversible enough and by making sure that the catalyst layer is stable. Consequently, kinetically meaningful Tafel slopes should ideally be obtained at high-mass-transport conditions, at relatively high reactant concentrations, and on a relatively low loading catalyst that has been preoxidized and has a constant amount of active sites. However, even ideal experimental conditions do not guarantee a horizontal Tafel slope region, as the potential-dependent Tafel slope might have a different origin, such as intermediate surface coverage or double layer effects.

Besides the OER, other reactions also exhibit a wide range of reported Tafel slopes for apparently similar catalysts and under apparently similar conditions. For example, for the alkaline HER, Tafel slopes of 91 mV/dec for Ru_2_O_2_/Co_3_O_4_ and 38 mV/dec for Ru/C_2_N have been reported in 1 and 0.1 M KOH, respectively; 113 mV/dec was found for a Pt disk, 59.9 mV/dec was reported for Pt/Fe on Ni foam, and 169, 131, and 114 mV/dec were found for Pt–Sm, Pt–Ho, and Pt–Ce, respectively.^39,40^ The kinetic meaningfulness of these Tafel slopes is not apparent. One of the effects described for alkaline HER is that it takes place at very negative potentials with respect to the pzc (potential of zero charge) of platinum, which causes a high concentration of cations to be in the double layer.^41^ This can cause significant effects of near-surface cation (over)crowding,^42^ which is not captured in Tafel kinetics. Theoretical considerations regarding cation-mediated reaction mechanisms and how they result in nonlinear Tafel slopes have been considered elsewhere.^43^ To investigate this effect, we applied the Tafel slope plot to the alkaline HER on a Pt disk RDE in 0.01 (+0.09 M NaClO_4_), 0.1, and 1 M NaOH.

Figure 3A shows that a higher NaOH concentration improves the activity; however, no horizontal Tafel slope region is observed in the Tafel slope plot in Figure 3B. What can be observed is a slower increase in Tafel slope value with increasing current density. Moreover, the low current region of the Tafel slope is not affected by the rotation rate from 500 to 2500 rpm, showing that this initial increase in the Tafel slope value is not due to bubble formation, while at higher current density, a clear rotation effect was observed (Figure S11). Additionally, the cation identity was studied, showing that Pt in 0.1 M LiOH is more active than KOH, as reported before (Figure S12).^42,44^ Similarly, no horizontal Tafel slope region was observed. Moreover, similar behavior was observed on the Pt microelectrode, which enables fast mass transport, in both LiOH and NaOH (Figure S13). Nevertheless, the Tafel slope plot shows that the increase in the apparent Tafel slope is less rapid for LiOH than for KOH/NaOH, similar to what was found for higher concentrations, which provides some insight into the potential limitations. However, kinetic information, such as the rate-determining step, cannot be directly obtained from these Tafel slope plots, at least not under the conditions used for these measurements. Interestingly, these Tafel slope plots do depend on the scan rate (Figure S14) in these nonhorizontal Tafel slope regions. In a previous work, we have related these deviations from ideal Tafel behavior to pH gradients in the interfacial diffusion layer.^43^

**Figure 3:**
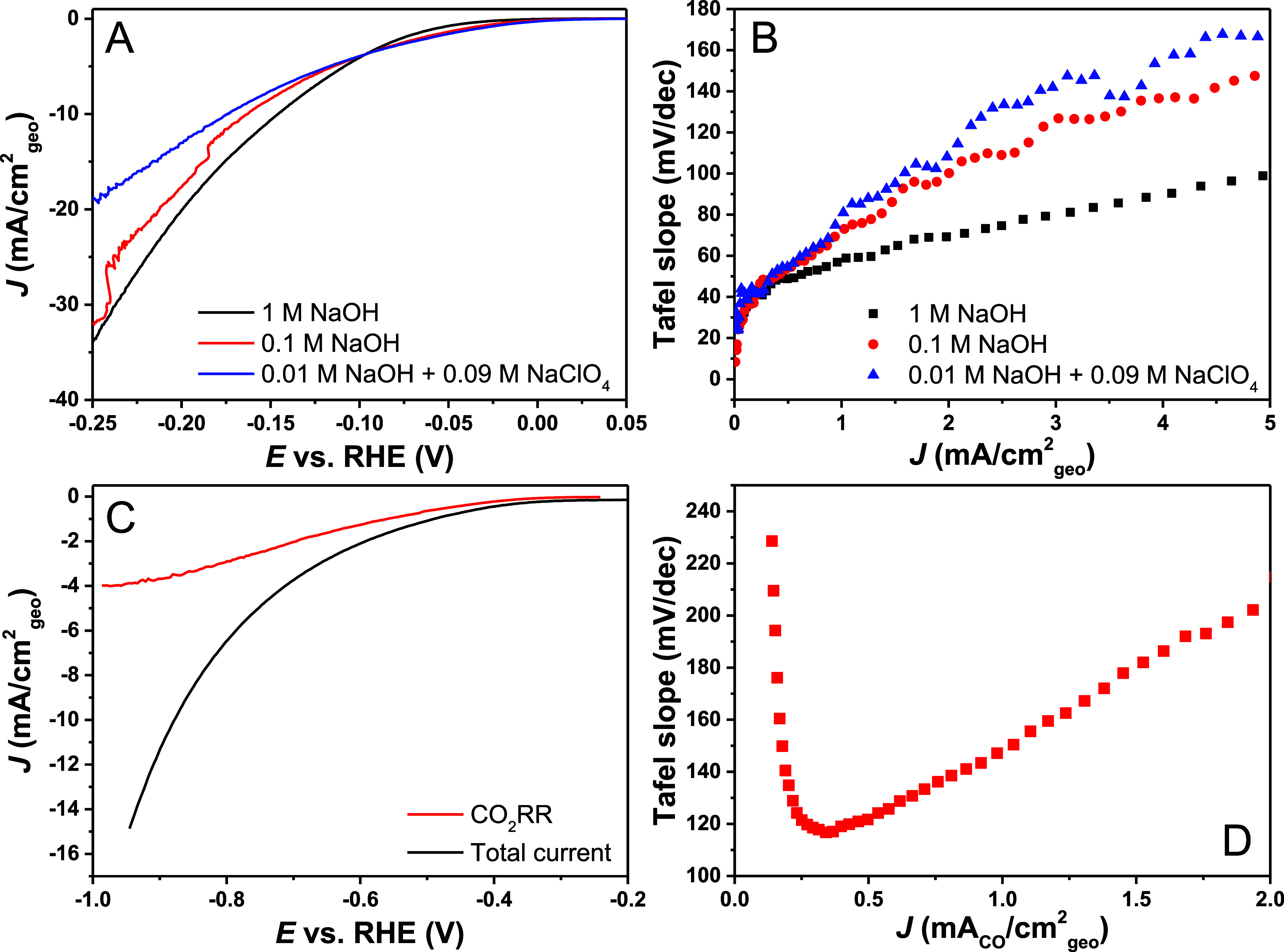
(A) LSVs at 2 mV/s of HER on polycrystalline Pt disk in a RDE setup in 0.01 NaOH + 0.09 M NaClO_4_, 0.1 and 1 M NaOH at 2500 rpm and (B) the corresponding Tafel slope plot vs current. Tafel slope plot vs potential is given in Figure S15. Alkaline HER results on the Pt microelectrode are shown in Figures S13 and S14. (C) LSV at 20 mV/s of the CO_2_ reduction to CO on a gold disk as measured by RRDE at 2500 rpm, given as both the full LSV and CO production as measured by the Au ring and (D) the corresponding Tafel slope plot obtained from the CO production. 85% ohmic resistance correction was done in situ and the remaining 15% manually afterward.

In addition to the alkaline HER, determining the Tafel slopes for the CO_2_ reduction reaction (CO_2_RR) to CO has also resulted in a large range of Tafel slope values in the literature. Tafel slope values have been reported from 59 mV/dec^45^ for oxide derived gold compared to 114 mV/dec^45^ for polycrystalline gold. In another study, 42 mV/dec was found for Au needles, compared to 80 mV/dec for Au rods and 96 mV/dec for Au particles.^35,36,46^ Often, the measured Tafel slope value is rounded off to the closest cardinal Tafel slope value.^47^ However, Bayesian data analysis for CO_2_ reduction showed no preference for any cardinal Tafel slope values.^47^ Improvements in Tafel slope determination have been attempted by providing a large near-surface concentration of CO_2_ with improved hydrodynamics. This indeed resulted in lower Tafel slope values.^48^ However, note that CO_2_ reduction is not limited by direct CO_2_ transport limitations but can be limited by the homogeneous reaction between OH^–^ and CO_2_ that occurs near the surface due to the production of hydroxide in both HER and CO_2_RR or to interfacial pH and corresponding cation gradients in general.^49^ Moreover, CO_2_RR is often further complicated by the possible production of a range of carbon products formed through multiple pathways with hydrogen as a byproduct,^12^ which must be disentangled.

We performed CO_2_ reduction to CO on gold, using a rotating ring disk electrode (RRDE) with a gold ring to disentangle CO production from hydrogen evolution,^50^ as shown in Figure 3C. In Figure 3D, it can be observed that perhaps there is an initial Tafel slope region around 120 mV/dec; however, even if that is true, this is only over a very small range. At larger overpotentials or higher current densities, there is a continuous increase in the Tafel slope value and no horizontal Tafel slope region. This shows that the Tafel slope determination of such a system is difficult, and no fundamental Tafel slope values were found under these conditions, which could explain the large range of Tafel slope values reported for the CO_2_RR to CO on gold, similar to alkaline HER. Because the experimental conditions combined with the potential/current range in which the Tafel slope is determined are important for the obtained value, they are a potentially arbitrary average of the values shown in Figure 3B,D. The advantage of the Tafel slope plot is that it shows when these limitations apply and if it makes sense to give a kinetic interpretation, for example, claim a certain rate-determining step based on Tafel slope analysis.

To investigate the Tafel slope behavior on a well-studied reaction that has (mostly) a consistent literature value, the Tafel slope plot was applied to acidic HER in 1 M HClO_4_. In Figure 4, it can be observed that the differential Tafel slope stays constant at ∼30 mV/dec from 2 to 15 mA/cm^2^_geo_ under an argon atmosphere. A Tafel slope of 30 mV/dec has been reported for many Pt catalysts in acid^51−55^ and the Tafel slope plot shows that this is also reasonably easy to obtain. It is interesting to note that the Tafel slope quickly becomes 30 mV/dec, and backward reactions do not play a very significant role, at least not above 2 mA/cm^2^_geo_ in argon. To investigate the dependence of the measured Tafel slope on the presence of a significant rate of the backward reaction, the Tafel slope plots of HER in hydrogen and argon atmospheres are compared (Figure S16). From that comparison, it can be observed that when there is a significant backward reaction, the Tafel slope is very much influenced, especially close to the equilibrium potential, which is of course not unexpected. Importantly, under a hydrogen atmosphere, the horizontal Tafel slope region at 30 mV/dec is strongly masked, whereas at higher overpotential the apparent Tafel slopes of course become similar in both argon and hydrogen atmosphere. This illustrates that kinetic data of a reversible reaction obtained near the equilibrium potential must be treated with great care.^56^ For comparison, a platinum microelectrode was used and a 30 mV/dec Tafel slope value was found for both H_2_SO_4_ and HClO_4_ (Figures S17 and S18), showing the consistency with which the 30 mV/dec Tafel slope can be observed for acidic HER on polycrystalline platinum for 2 different systems in 2 different electrolytes, as clearly observed in the Tafel slope plots.

**Figure 4:**
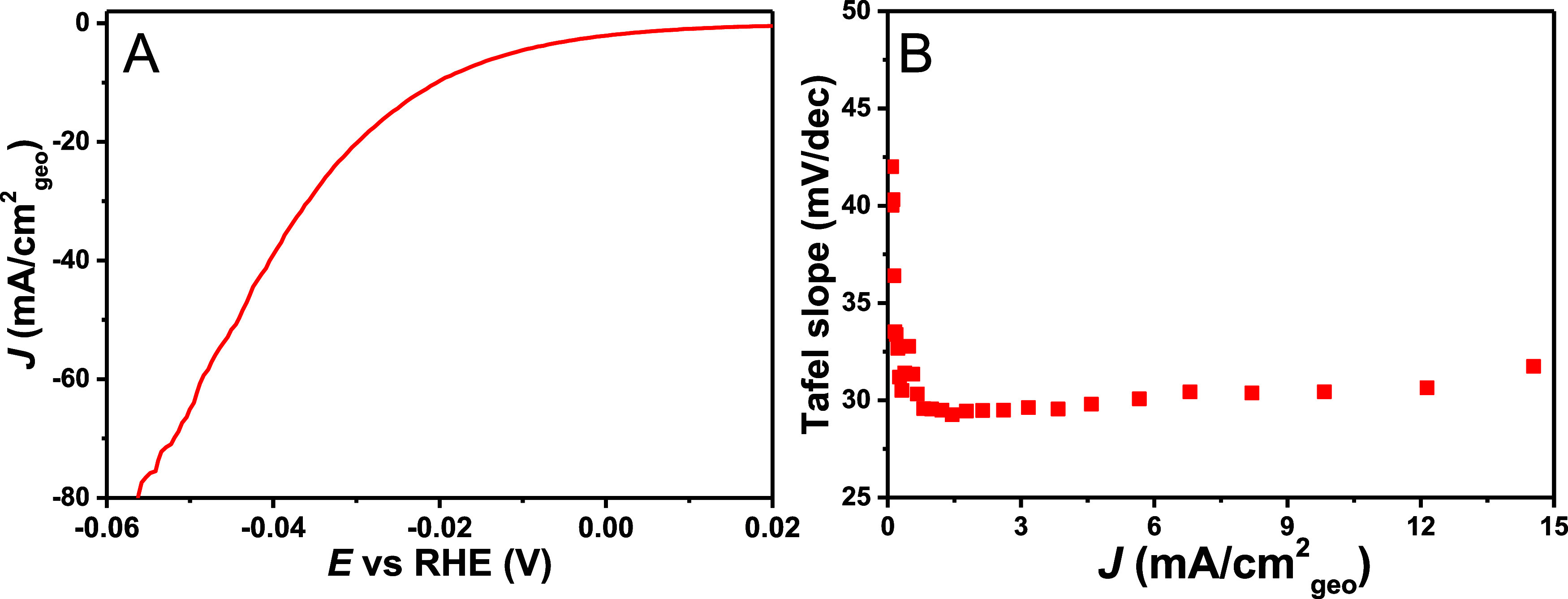
(A) LSV at 2 mV/s of acidic HER polycrystalline on Pt disk in a RDE setup in 1 M HClO_4_ at 2500 rpm and (B) the corresponding Tafel slope plot vs current density, showing a horizontal Tafel slope region of 30 mV/dec (Tafel slope plot vs potential is given in Figure S19). 85% ohmic resistance correction was done in situ and the remaining 15% manually afterward. Similar values were observed for the Pt microelectrode in both HClO_4_ and H_2_SO_4_ (Figures S17 and S18).

It is possible to identify a horizontal Tafel slope region over a larger range using a Tafel slope plot, as shown for the acidic HER on Pt in HClO_4_ and the OER on NiFeOOH. However, for other reactions (e.g., alkaline HER on Pt and CO2RR to CO on Au), such regions were not found under commonly used conditions. For CO_2_RR, this might be possible if high surface CO_2_ concentrations are realized.^37^ However, it is not a given that the requirements for Tafel analysis will be obtained for the investigated system. The application of the Tafel slope plot with different mass transport conditions and, if relevant, different loadings could be a powerful tool to show that such conditions are (not) obtained. The use of more complex models that can fit nonlinear Tafel slopes would be necessary to gain kinetic insight from intrinsically nonlinear Tafel slope plots.

A complementary method in which the Tafel slope is not fitted in an arbitrary potential window is using Bayesian analysis.^47^ Bayesian analysis generates a statistical distribution of Tafel slopes from the current potential curve. Watkins et al. have used Bayesian analysis to study the effect of cell geometry on the observed Tafel slope during CO_2_RR on copper, also showing the importance of having the experimental (hydrodynamic, in their case) conditions right for obtaining (kinetically meaningful) Tafel slopes.^37^ By comparison, the Tafel slope plot displays the change in the Tafel slope as a function of potential or current, which we believe provides an intuitive and easy way to see if a cardinal Tafel slope value is involved. Also, the potential dependence on the Tafel slope itself contains, in principle, meaningful information, which is not immediately evident from the Bayesian analysis. So, the Tafel slope plot, or similar numerical differentiation approaches, provides a simple and intuitive method to obtain kinetic insight by indicating the kinetic meaningfulness of the Tafel slope value or by preventing overinterpretation of the Tafel analysis.

In conclusion, to improve fundamental kinetic insights, it is essential to determine a region in which the Tafel slope is not limited by nonkinetic effects, as evidenced by a horizontal region in the Tafel slope plot. For the OER on NiFeOOH in 0.2 M KOH, we show that under high-mass-transport conditions at low loading similar Tafel slope values are obtained in this horizontal Tafel slope region using potentiodynamic, potentiostatic, and impedance spectroscopy-based techniques. Moreover, the Tafel slope plot was applied to CO_2_RR on Au and alkaline HER on Pt at different NaOH concentrations. For both reactions, no horizontal Tafel slope region was observed, which explains the wide range of Tafel slopes reported in the literature for these reactions. This is because the measurement conditions combined with the potential range in which the Tafel slope was determined are major contributors to the obtained value, and the reported value will then be a potentially arbitrary average of the values found in that specific potential region. In contrast, the acidic HER in 1 M HClO_4_ on Pt shows a horizontal Tafel slope value of 30 mV/dec over a relatively large current density/potential region, which is also the often-reported literature value. More complex models that can fit the potential-dependent Tafel slope would be required to obtain additional insight when the deviation from linearity is not a result of the experimental conditions used but rather an intrinsic property of the studied system. Therefore, to confirm the kinetic meaningfulness of the Tafel slope value, a horizontal region should preferably be observed in the Tafel slope plot for different mass transport conditions and, if relevant, different loadings or reactant concentrations. Moreover, the obtained Tafel slope values can be compared to those of potentiostatic techniques such as CA and impedance spectroscopy and should ideally result in the same value. By application of the Tafel slope plot, or similar numerical differentiation, Tafel slope values can be compared more fairly, and it is our expectation that similar Tafel slope values will then be observed for alike materials when measured under similar conditions and/or that it will prevent overinterpretation when no consistent Tafel region will be observed at all.

## Experimental Section

### General Cleaning Procedure

Glassware and plastic cells were stored in 0.1–0.5 M H_2_SO_4_ (95–98%, ACS reagent, Sigma-Aldrich) solution containing 1 g/L KMnO4 (>99%, ACS reagent, Emsure). Then, the glassware was cleaned in diluted piranha solution (H_2_O_2_, 35%, Merck and H_2_SO_4_, 95–98%, ACS reagent, Sigma-Aldrich) and boiled in milli-Q water (resistance: 18.2 MΩ•cm) at least 3 times.

### Oxygen Evolution Reaction, RDE Experiments

A rotating disk electrode (RDE) setup was used (MSR rotator, Pine research). The substrate for the working electrode was a fixed gold disk in a PEEK sheath in a shaft to achieve increased rotation rate (E2MPK FastSpeed RDE, gold disk: 5 mm OD, 0.196 cm^2^, Pine research). First, the gold disk was polished on a microcloth (Buehler) with a diamond suspension of 3, 1, and 0.25 μm (MetaDi, Buehler) respectively. Thereafter, the tip was sonicated for at least 10 min to remove attached diamond particles.

Prior to deposition, the real gold surface area was checked in a three electrode cell, with the RDE as working electrode, a platinum counter electrode, and a large hydroflex reversible hydrogen reference electrode (Gaskatel). CV scans were conducted in the range 0.05–1.75 V vs RHE at 50 mV/s in 0.1 M H_2_SO_4_ (96%, suprapur, Merck). To deposit the NiFe catalyst precursor, the follow deposition conditions were used: 1.4 mA for 5 s with a constant rotation rate of 400 rpm in a 80 mM Ni(NO_3_)_2_·6H_2_O (99.99% trace metal basis, Sigma-Aldrich) + 20 mM FeSO_4_·7H_2_O (>99%, ACS reagent, Sigma-Aldrich) solution in Milli-Q water. The deposition electrolyte was prepared from a 80 mM Ni(NO_3_)_2_.6H_2_O stock solution to which FeSO_4_·7H_2_O salt was added after purging with argon for at least 15 min, to prevent FeO_*x*_ formation.

For the oxygen evolution reaction, a three-electrode setup with a rotator was used, with the deposited NiFe layer on the Au disk as described above. To prevent glass dissolution into the electrolyte a plastic (Nalgene) cell was used,^57^ a Hydroflex RHE electrode (Gaskatel) was used as reference electrode and a large surface area gold wire (99%, 0.8 mm thick, Mateck) was used as the counter electrode. The 1 M stock solution was prepared from KOH pellets (99.99% semiconductor grade, 15% water, Sigma-Aldrich) that were made iron-free with the method described before,^58^ whereby the iron was scavenged by dispersed Ni(OH)_2_ in the 1 M XOH electrolyte in a centrifugal tube, and then the electrolyte was prepared by centrifuging at 6000 rpm, after which the required amount of electrolyte was transferred to the plastic cell by a pipet. A 0.2 M KOH solution was prepared from this stock. Prior to the Tafel slope measurements, the catalyst layer was activated by 50 scans at 50 mV/s between 1.2 and 1.6 V vs RHE (CV, 85% *iR* correction).

For the oxygen evolution reaction, first a CV–CA–LSV procedure was performed to remove the majority of the Ni oxidation contribution at low current densities. The CV was taken at 10 mV/s from 1.2 V vs RHE to 1.550 V vs RHE and back to 1.455 V vs RHE, and then a CA was performed for 10 s at 1.455 V vs RHE. Finally, a LSV, in which the potential is swept over a preset potential region and the current is measured, was performed at 2 mV/s, or other scan rate as specified, from 1.455 to the desired final potential; ohmic resistance was determined from the impedance measurement and compensated for 85% in situ and for 15% manually afterward to prevent inducing resonance.

The impedance spectroscopy measurements were performed at different potentials from 50 kHz to 1 Hz with a 10 mV amplitude from 1.42 to 1.65 V vs RHE (*iR* corrected afterward) in 25 steps, as given in Figure S5. The charge transfer resistance was determined by fitting an equivalent circuit containing a solution resistance in series with a parallel CPE and charge transfer resistance. During the fit, the parameter *R*_ohm_ was fitted between 18 and 20 ohm, *R*_ct_ between 1 and 100 ohm, the CPE value between 5 × 10^–6^ and 5 × 10^–3^, and the CPE exponent between 0.7 and 1.0. Moreover, chronoamperometry was used, in which the potential is set and the resulting current is measured as a function of time (chronopotentiometry or CP could also be used but does the opposite: a current is set and the potential is measured). The current density after 1 min of stabilization time was used for Tafel analysis (prior to the impedance measurement itself). The potential was *iR* corrected for 100% afterward with the ohmic resistance that was obtained from impedance measurements (Figure S6), as the intersection between the real axis at high frequency. Impedance spectra were analyzed with the EIS spectrum analyzer (ABC chemistry, by Bandarenka and Ragoisha^59^).

The Tafel slope analysis was performed like described in our previous work.^14^ The Tafel slope was determined from LSV over small potential ranges of 10 mV for the OER and HER and 20 mV for the CO2RR (due to a lower number of available data points in the RRDE measurement) and plotted against the average current or potential. In such a plot it can be observed how the (apparent) Tafel slope increases at increasing current density and to which value it converges at low current density. A schematic overview of the considerations for Tafel analysis and plotting the Tafel slope plot are given in Figure 1 and Figure S1. The Tafel slope formula is given as
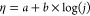
1with η the overpotential in mV, *a* the exchange current density, *j* the current density mA/cm^2^, and *b* the Tafel slope in mV/dec.

And for impedance this is
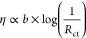
2with η the overpotential in mV, *R*_ct_ the charge transfer resistance, and *b* the Tafel slope in mV/dec. The Tafel slope is then obtained from the linear fit of the overpotential vs log *J* or from the overpotential vs log 1/*R*_ct_ for LSV (CA/CP) and impedance spectroscopy, respectively.

### Hydrogen Evolution Reaction, RDE Experiments

A rotating disk electrode (RDE) setup was used (MSR rotator, Pine research). As the insert, a Pt disk (5 mm OD; 4 mm thick; 0.196 cm^2^, Pine research) was used. For experiments in acid, a glass cell was used with HClO_4_ (60%, ACS reagent, Emsure, Merck) or H_2_SO_4_ (96%, Suprapur, Merck). For the base, a Nalgene cell was used with LiOH·H_2_O (>99.95% trace metals basis, Sigma-Aldrich), NaOH (30% solution, Suprapur, Merck), and KOH (>99.99% trace metal basis, 15% water, semiconductor grade, Sigma-Aldrich) and a counter electrode from Pt wire (99.9%, Mateck) or gold wire (99.9%, Mateck).

### CO_2_ Reduction Reaction, RRDE Experiments

CO_2_ reduction experiments were performed using a RRDE setup as described elsewhere.^60^ In short, the Au disk and ring were mechanically polished followed by a dopamine modification (dopamine hydrochloride, ≥98.5%, Sigma-Aldrich) of the Teflon spacer. After the removal of the dopamine from the Au electrodes, the RRDE tip was ready for use. The CO_2_ reduction measurements were performed in CO_2_ (4.5 purity, Linde) saturated 0.1 M NaHCO_3_ (≥99.7% Sigma-Aldrich) electrolyte at 2500 rpm. The disk was scanned from +0.05 to −1.0 V vs RHE with a scan rate of 20 mV/s and the ring was set to 1.0 V vs RHE. The collection efficiency of the ring was determined after the experiment by the use of a ferrocyanide couple (K_3_Fe(CN)_6_, >99%, Sigma-Aldrich).

### Pt Microelectrode

The Pt microelectrode was fabricated by sealing a 100 μm Pt wire (99.99%, Goodfellow) within a soda-lime glass capillary (Hilgenberg) using a butane torch. After fabrication, the Pt microelectrode underwent a polishing procedure using a microcloth (Buehler) with diamond particle suspensions of decreasing particle sizes (3, 1, 0.25, and 0.05 μm, Buehler). The Pt microelectrode was sonicated in DI water to remove any residual particles from its surface. Lastly, the Pt microelectrode was electrochemically cleaned by cycling between 0.05 and 1.35 V vs RHE (1 V/s, 30 times) in 1 M H_2_SO_4_. No *iR* correction was applied to the data obtained with the Pt microelectrode in the 1 M acid solution, given its low current levels. *iR* correction was applied in the base from the ohmic resistance determined from impedance spectroscopy.

## Supporting Information Available

Additional details for plotting the Tafel plot (Figure S2), additional experiments for what influences obtained Tafel slopes for OER (Figures S1 and S3–S10) and HER (Figures S11–S19), table of *iR* corrected potential, current density, charge transfer resistance and corresponding error % and CPE value and exponent (Table S1) (PDF)
