# Supplementary material for: Tafel Slope Plot as a Tool to Analyze Electrocatalytic Reactions
Source: ACS Energy Lett. 2024 Apr 1;9(4):1871–9. doi: 10.1021/acsenergylett.4c00266 (PMC11019648; doi:10.1021/acsenergylett.4c00266)
Supplement: Supplementary file 1 — nz4c00266_si_001.pdf [file nz4c00266_si_001.pdf]

Supporting information to:

Tafel Slope Plot as a Tool to Analyse Electrocatalytic Reactions

Onno van der Heijden, Sunghak Park, Rafaël Vos, Jordy J.J. Eggebeen, Marc T.M. Koper

Leiden Institute of Chemistry, Leiden University, 2333 CC, Leiden, the Netherlands.

Contents

OER: determining ohmic resistance in the presence of bubbles 2
Plotting Tafel slope plots 3
Tafel plot of CA, EIS and LSV, extended region 4
OER: LSV & CA 5
OER: impedance spectroscopy 6
OER: impedance spectroscopy: ohmic resistance 7
Tafel plot with different intervals 8
OER: scan rate dependence, LSV with Ni redox 9
OER: scan rate dependence, CA – CV – LSV 10
Alkaline HER on RDE: rotation rate dependence 11
Alkaline HER on RDE: 0.1 M KOH & LiOH 12
Alkaline HER on Pt microelectrode: 0.1 M NaOH & LiOH 13
Alkaline HER on RDE: 0.01 M – 1 M NaOH on Pt 14
Acidic HER on RDE: 1 M $HClO_4$, Ar compared to H_2 atmosphere 15
Acidic HER on Pt microelectrode: HER in $HClO_4$ 16
Acidic HER on Pt microelectrode: 1 M H_2SO_4 17
Acidic HER on Pt microelectrode: Tafel slope plot vs. potential on 1 M $HClO_4$ 18
References 19

OER: determining ohmic resistance in the presence of bubbles

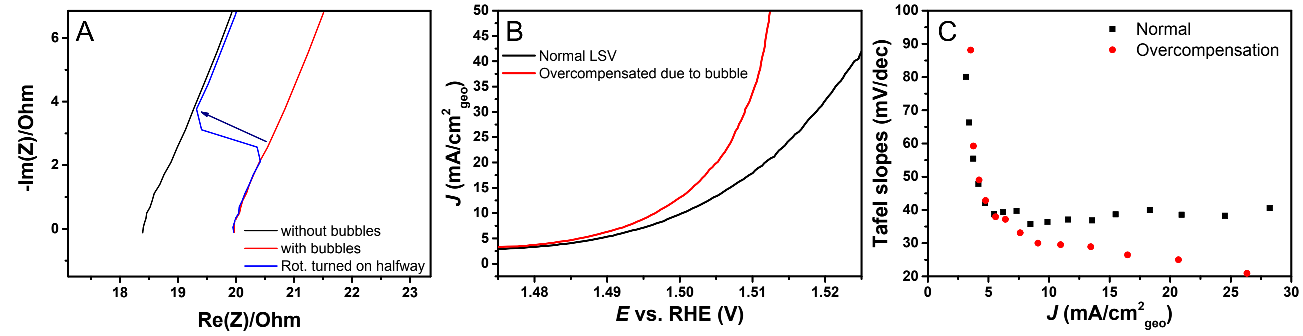

Figure S1. (A) impedance spectrum with bubbles on the surface, then during removal during the impedance measurement (by increasing rotation 0 → 3000 RPM) and finally after removal, (B) LSV (with Ni redox) with iR compensation based on ohmic resistance as measured (red) with bubbles and (black) without bubbles, (C) corresponding Tafel slope plot, showing how ohmic resistance determined with bubbles on the surface might result in too low Tafel slope values due to overcompensation (high Tafel slope values at $J < 5$ mA/cm^2 are due to Ni oxidation contribution).

Plotting Tafel slope plots

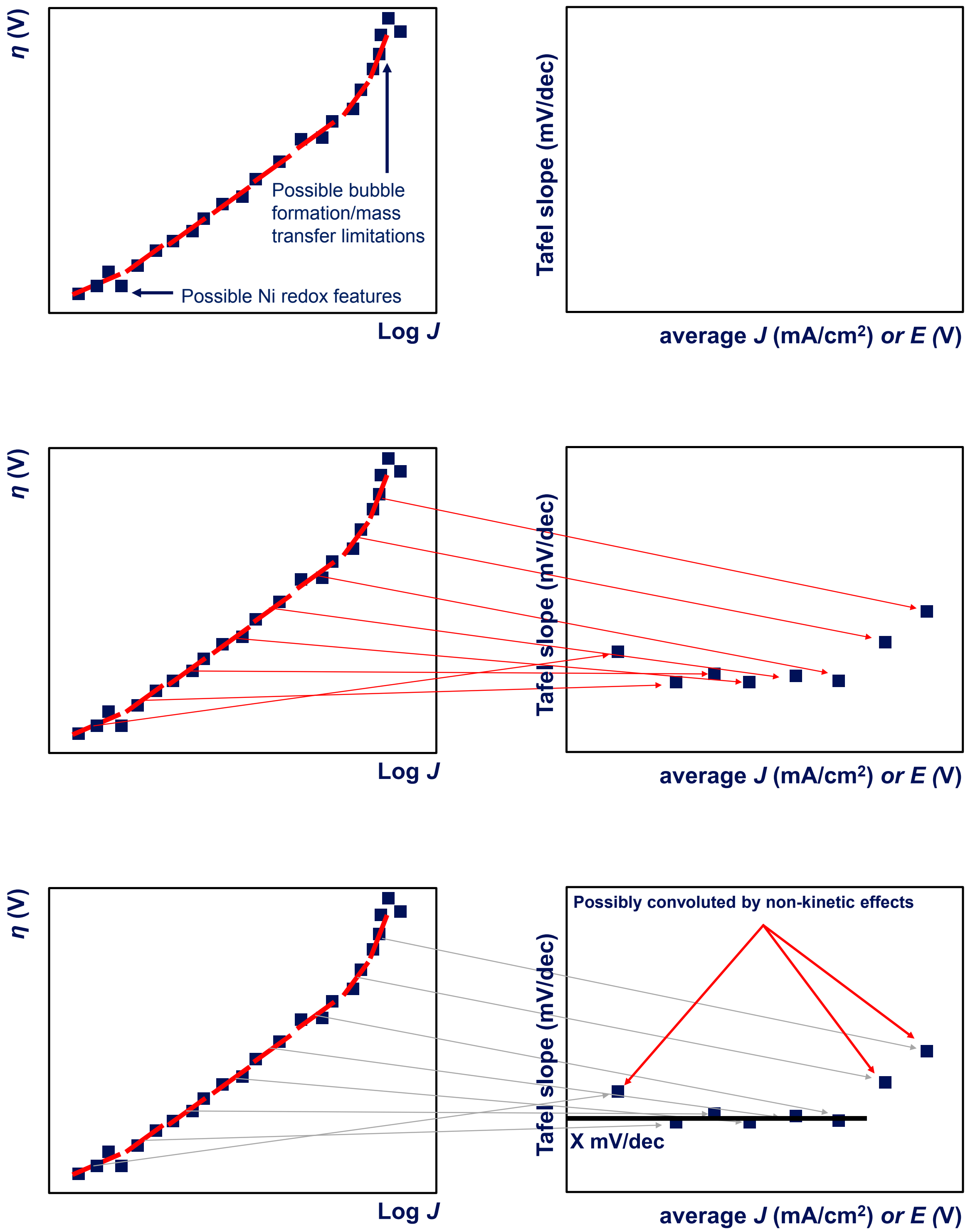

Figure S2. Schematic representation of how to construct the Tafel slope plot, with horizontal regions indicating potentially kinetically meaningful values.

Note: The slopes can be taken in overlapping potential intervals (e.g. 1.45 – 1.46 V; 1.455 – 1.465 V), it is important that the obtained Tafel slope does not depend on the chosen mV range, as is further explored in Figure S8.[1]

Tafel plot of CA, EIS and LSV, extended region

To show how the Tafel slope values change outside the horizontal region in the Tafel slope plot the LSV, CA and EIS measurements are shown below with the line extrapolated from the fit from Figure 2 in the main text.

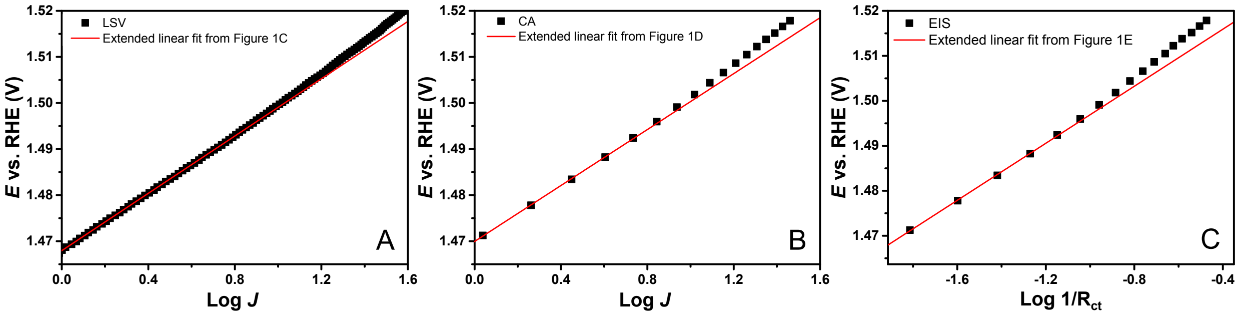

Figure S3. (A) LSV over extended region, the line is extrapolated from the region as fitted in Figure 2C, (B) CA over extended region, the line is extrapolated from the region as fitted in Figure 2D, (C) EIS over extended region, extrapolated from the fit of Figure 2E. The Tafel slope values start to deviate at a similar current density for LSV, CA and EIS for this system.

OER: LSV & CA

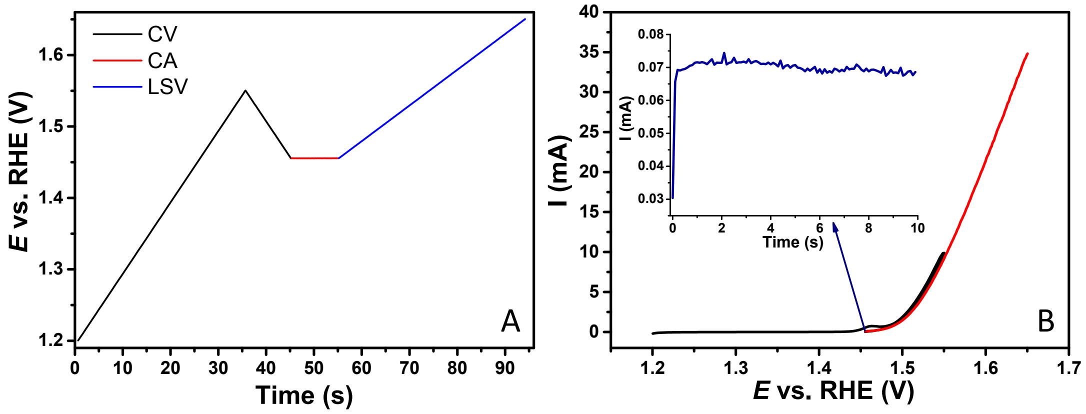

Figure S4. CV – CA – LSV method to remove the majority of the Ni oxidation contribution in (A) potential vs. time and (B) as current vs potential.

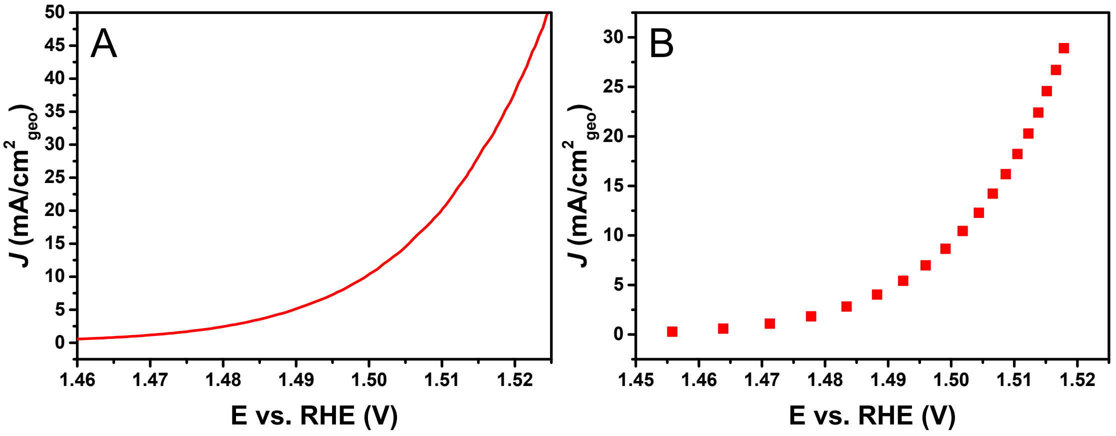

Figure S5. (A) LSV with removed Ni redox (CV-CA-LSV as shown in Figure S4), (B) CA with current density determined after 1 minute for each potential point.

OER: impedance spectroscopy

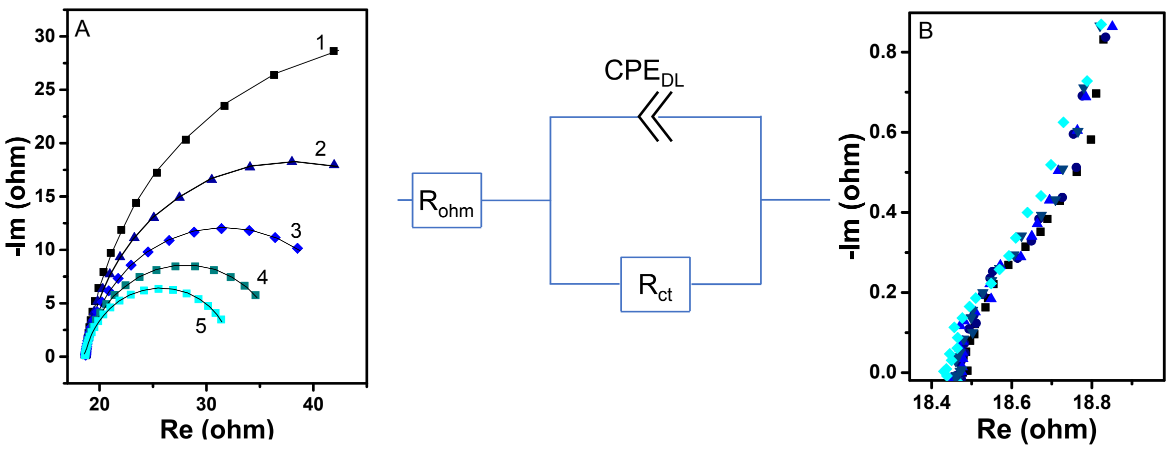

Figure S6. A) Impedance plots used for determining the Tafel slope from the charge transfer resistance fitted from 500 to 1 Hz, with the corresponding fits with the RC circuit given in the middle, as fitted with EIS Spectrum Analyser (ABC chemistry, by A. Bandarenka & G. Ragoisha). RC circuit consists of an ohmic resistance (R_{ohm}) in series with a constant phase element (CPE) representing the double layer capacitance in parallel with the charge transfer resistance (R_{ct}). B) At higher frequencies there is also a smaller semi-circle that is related with the layer and loading[2]. Because we do not fit that region the ohmic resistances given in Table S1 are slightly too high. The correct values, as given by the intersection between the imaginary and real axis, are given in Figure S6.

Table S1. 100% manually iR corrected potential, current density, charge transfer resistance and corresponding error% (relative estimated error in %) and CPE value and exponent for the Tafel slope determination with impedance spectroscopy.

Nr	E vs. RHE (V)	J (mA/ cm^2_{geo})	R_{ohmic} (ohm)	Error%	R_{ct} (ohm)	Error%	CPE value (p)	Error%	CPE exponent (n)	Error%
1	1.475	1.09	18.7	0.5	65.2	2.4	0.0037	1.0	0.95	0.3
2	1.484	1.83	18.7	0.4	39.6	1.7	0.0037	1.1	0.95	0.3
3	1.494	2.81	18.7	0.4	26.2	1.6	0.0036	1.3	0.94	0.4
4	1.503	4.02	18.7	0.4	18.6	1.5	0.0036	1.4	0.94	0.4
5	1.512	5.42	18.6	0.3	14.1	1.6	0.0036	1.6	0.94	0.4

The Tafel slope can then be calculated from the linear fit of the overpotential vs. log $1/R_{ct}$.

OER: impedance spectroscopy: ohmic resistance

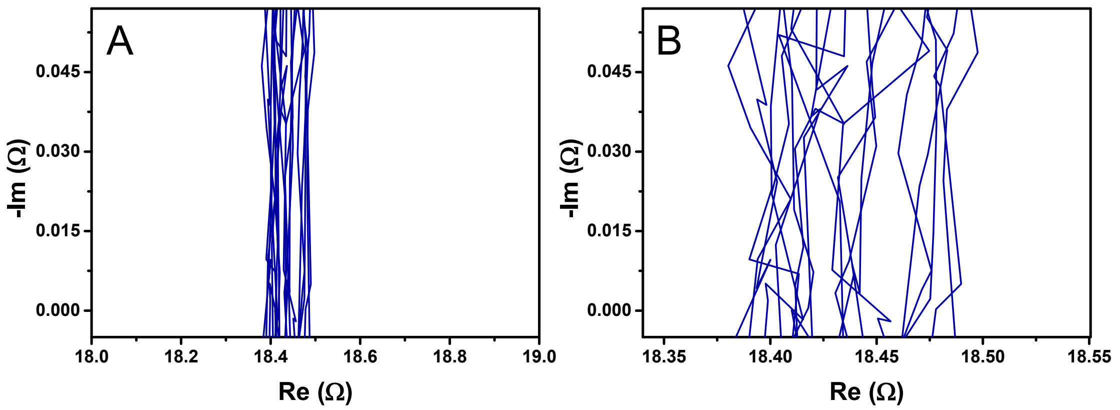

Figure S7. Ohmic resistance measured at different potentials at the intersect with the real axis between 1.42 and 1.65 V vs. RHE (no iR compensation) in 0.2 M NaOH (to ~30 mA/cm^2), (B) zoomed in showing only a very small spread from 18.38 - 18.50 ohm. So, a very stable ohmic drop was observed at increasing potential at 4000 RPM.

Tafel plot with different intervals

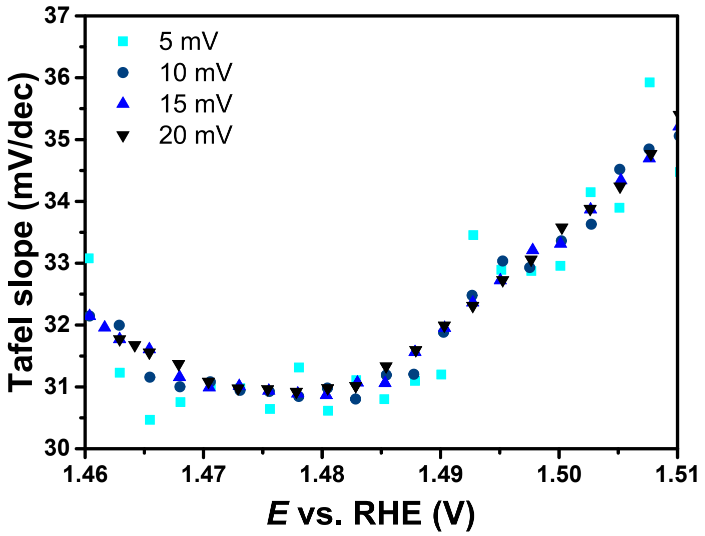

Figure S8. Tafel slope plot obtained from different potential windows of 5, 10, 15 and 20 mV, to show how the chosen potential window over which the Tafel slope is determined, affects the resulting Tafel slope plot. Smaller windows appear to show a larger horizontal region, as more data points will be fully within the `Tafel` region (same kinetic regime). However, the smaller interval also shows more noise, as it is taken over fewer data points. Optimization of the range, while making sure the obtained Tafel slope value is the same in the horizontal region, will be necessary for different data sets, and clearly a high density of data points is preferred.

OER: scan rate dependence, LSV with Ni redox

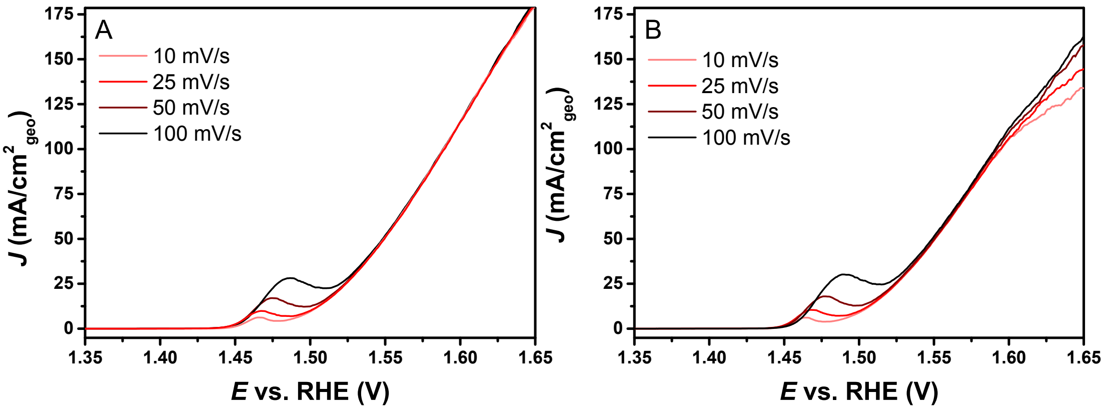

Figure S9. (A) Scan rate dependence for LSV without pre-oxidation at high mass transport conditions (4000 RPM), (B) LSV without pre-oxidation at low mass transport conditions (1000 RPM), showing a scan rate dependence at higher current density at lower mass transport conditions (85% ohmic resistance correction, but the ohmic resistances are the same for the measured LSVs).

OER: scan rate dependence, CA – CV – LSV

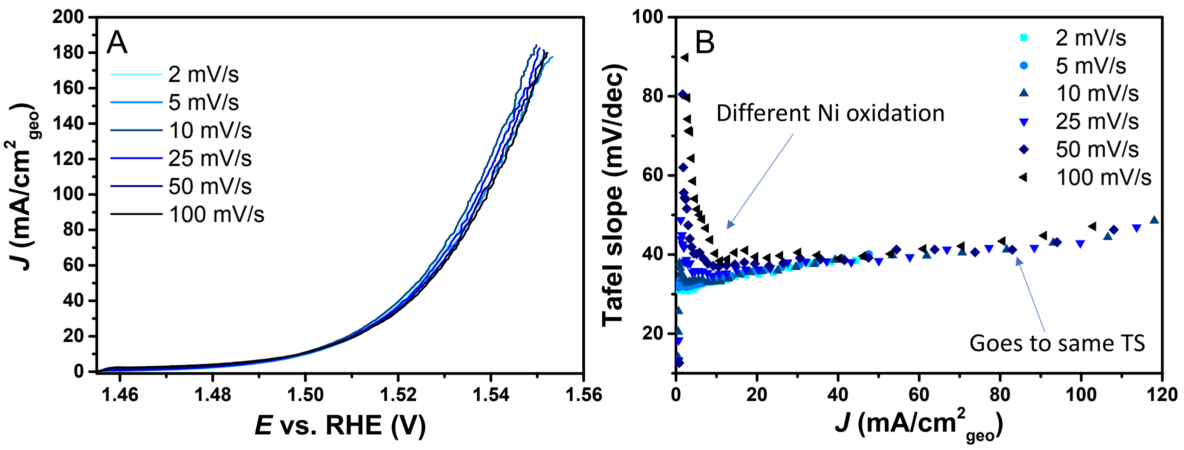

Figure S10. Scan rate dependence of the (A) LSV after the CA – CV – LSV method to remove the majority of the Ni redox contribution, (B) corresponding Tafel slope plot, showing no scan rate dependence at higher current densities after pre-oxidation treatment and at high rotation rate, but still some influence due to some catalyst oxidation processes at low current density. So it is still advisable to use very low scan rates < 5 mV/s.

Alkaline HER on RDE: rotation rate dependence

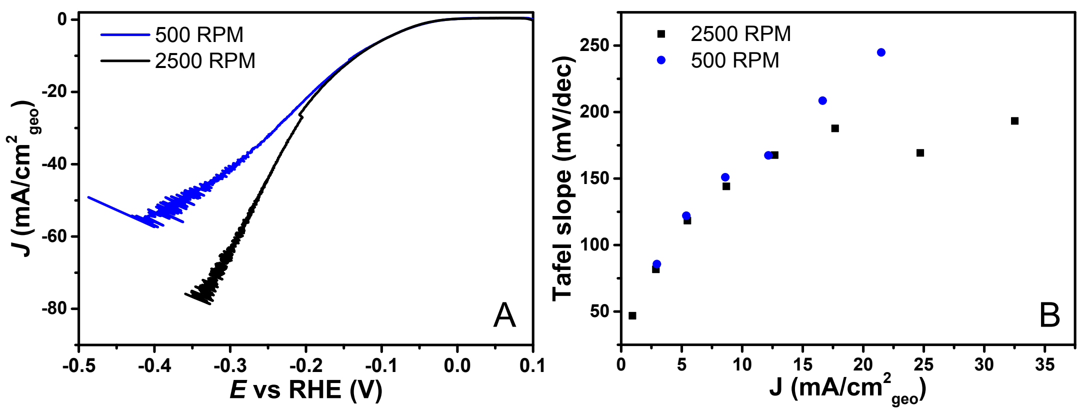

Figure S11. (A) LSV of alkaline HER on Pt at 500 and 2500 RPM in 0.1 M KOH, (B) corresponding Tafel slope plot. This shows the rotation dependence of alkaline HER on Pt disk RDE only happens at higher current density and the initial increase in Tafel slope is not due to bubbles.

Alkaline HER on RDE: 0.1 M KOH & LiOH

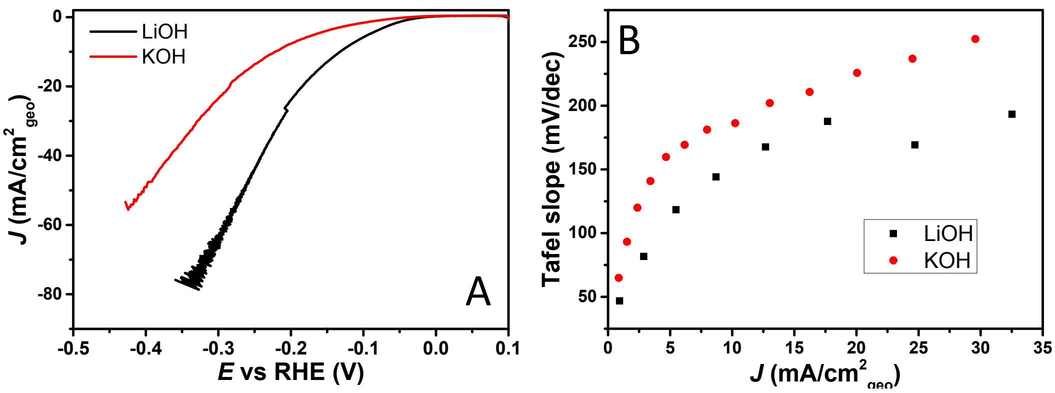

Figure S12. (A) LSV of alkaline HER on Pt in 0.1 M LiOH and 0.1 M KOH, (B) corresponding Tafel slope plot. While no clear horizontal regions can be found in either electrolyte, for KOH the Tafel slope value increases faster compared to LiOH.

Alkaline HER on Pt microelectrode: 0.1 M NaOH & LiOH

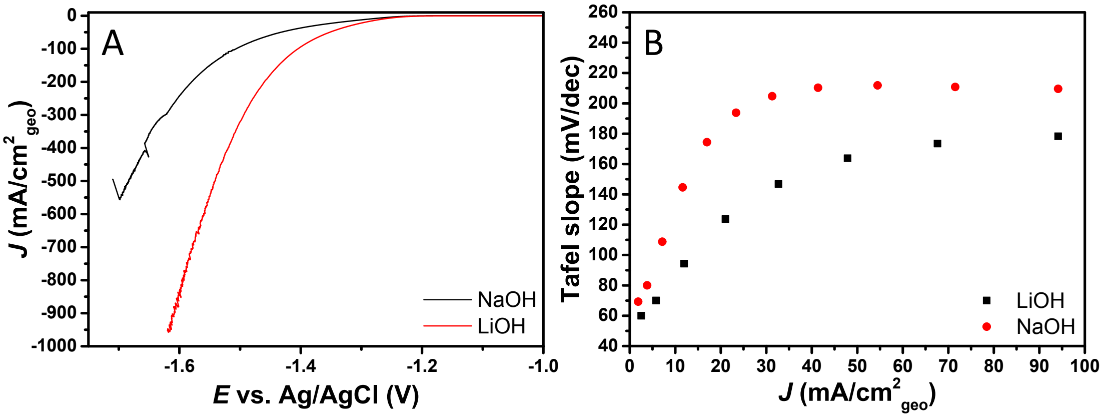

Figure S13. (A) LSV of Pt microelectrode in 0.1 M NaOH and LiOH, (B) corresponding Tafel slope plot, showing a fast increase in Tafel slope for both, but slower with LiOH, similar to RDE. The ohmic resistance is hard to determine on the Pt microelectrodes, as the wire is thin and long resulting in a feature at high frequency related to that. Therefore, a slight overcompensation might exist at higher current density, however low current density measurements are not strongly affected by this due to very low currents.

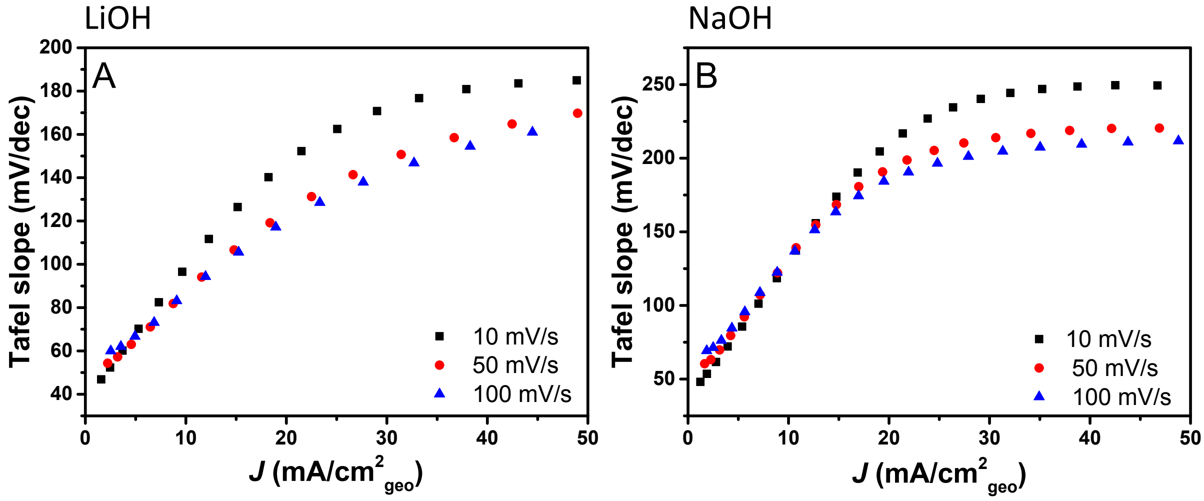

Figure S14. (A) Tafel slope plot of HER in 0.1 M LiOH on a Pt microelectrode as a function of scan rate, (B) Tafel slope plot of HER in 0.1 M NaOH on a Pt microelectrode as a function of scan rate, showing a scan rate dependence for this system.

Alkaline HER on RDE: 0.01 M – 1 M NaOH on Pt

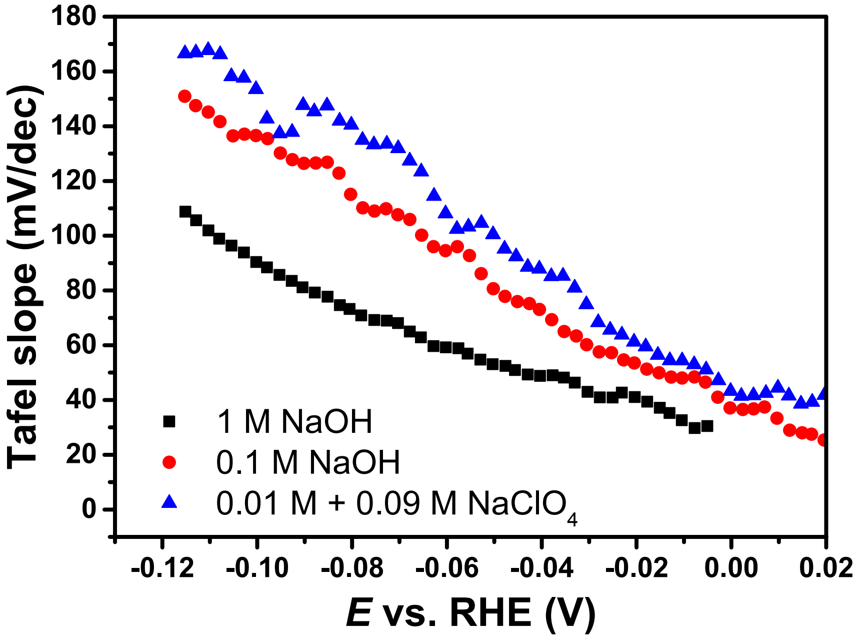

Figure S15. (A) Tafel slope plot vs. potential in 1 M NaOH, 0.1 M NaOH and 0.01 M NaOH + 0.09 M $NaClO_4$ vs. potential on the RDE setup.

Acidic HER on RDE: 1 M $HClO_4$, Ar compared to H_2 atmosphere

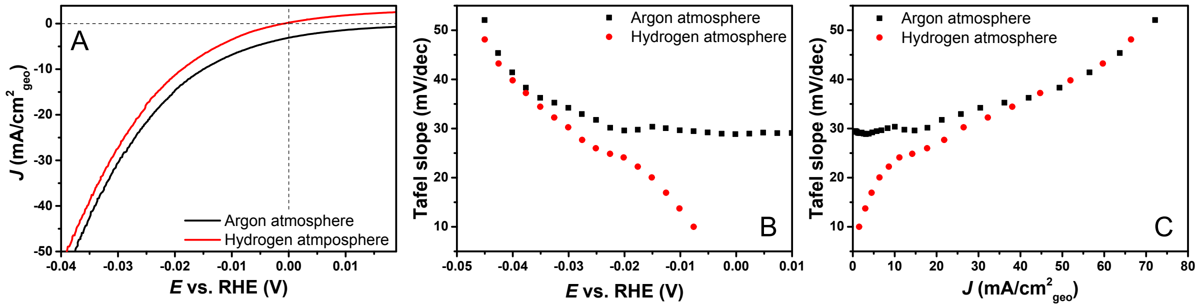

Figure S16. (A) LSV at 2 mV/s of a Pt disk electrode in a RDE setup at 2500 RPM in 1 M $HClO_4$ under argon and hydrogen atmosphere, (B) corresponding Tafel slope plot vs. potential, (C) corresponding Tafel slope plot vs. current density. What can be observed from this plot is that under hydrogen atmosphere there is no horizontal Tafel region of 30 mV/dec and that at low overpotential Tafel analysis on the hydrogen saturated system will result "mixed" Tafel slopes (i.e., < 30 mV/dec) due to H_2 oxidation (the backward reaction), which obviously becomes more pronounced closer to the equilibrium potential. This observation has also been made for Pt/C systems with different loadings which exhibit hydrogen reoxidation due to high hydrogen concentrations remaining within the structure[3].

Acidic HER on Pt microelectrode: HER in $HClO_4$

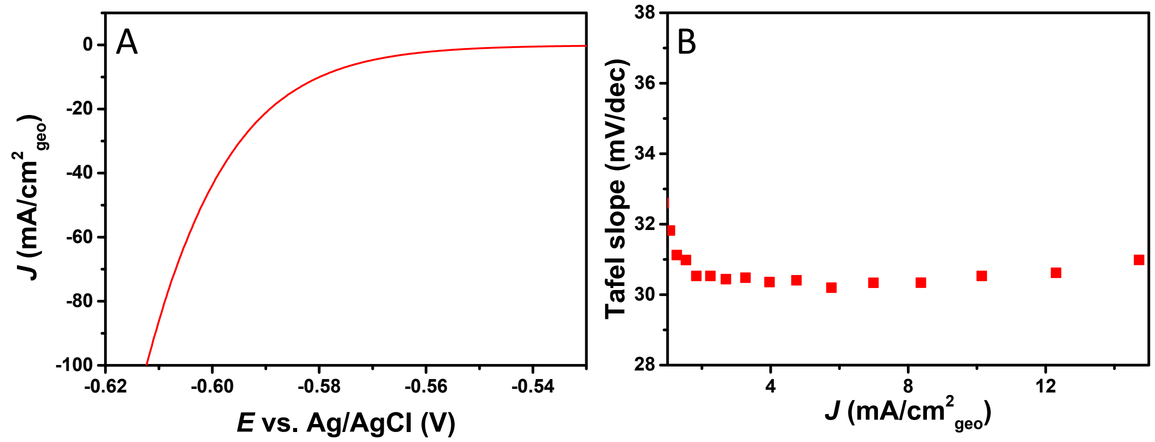

Figure S17. (A) LSV of HER on Pt microelectrode in 1 M $HClO_4$, (B) corresponding Tafel slope plot of the Pt microelectrode in 1 M $HClO_4$ vs. current density, showing a relatively large horizontal region similar to the RDE.

Acidic HER on Pt microelectrode: 1 M H_2SO_4

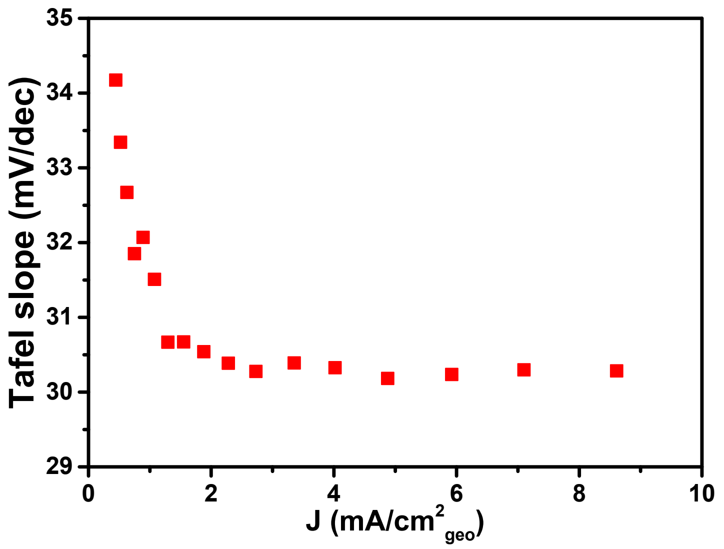

Figure S18. Tafel slope plot for acidic HER in H_2SO_4, showing how the same Tafel slope value of 30 mV/dec is observed for the RDE setup.

Acidic HER on Pt microelectrode: Tafel slope plot vs. potential on 1 M $HClO_4$

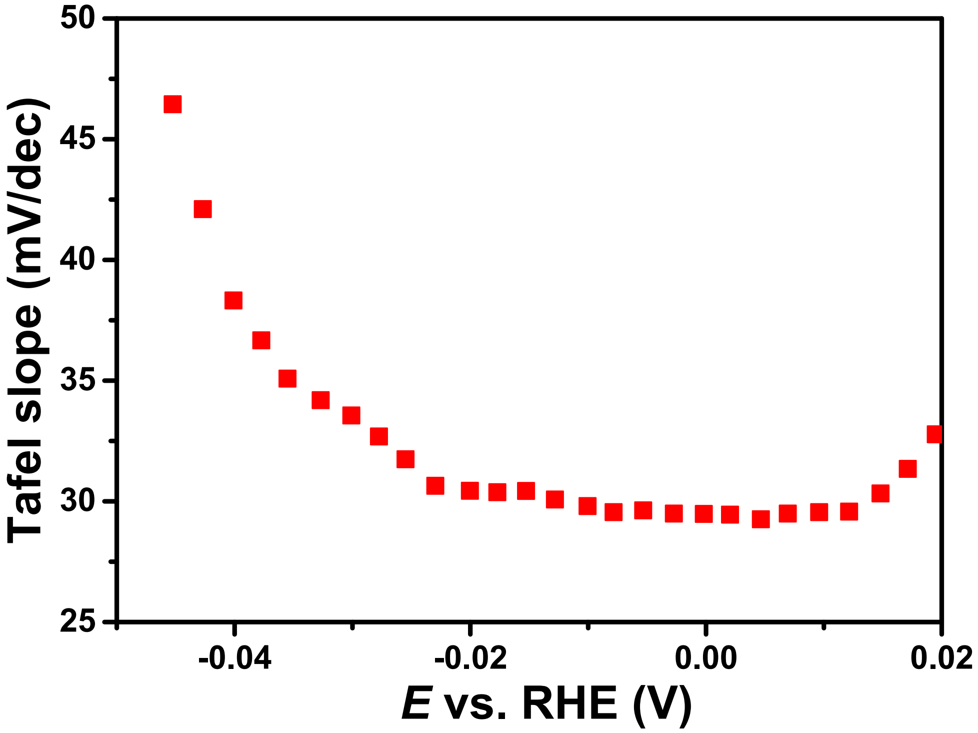

Figure S19. Tafel slope plot vs. potential for Pt HER in 1 M $HClO_4$ on the RDE setup.

References

(1) Heijden, O. van der; Park, S.; Eggebeen, J. J. J.; Koper, M. T. M. Non-Kinetic Effects Convolute Activity and Tafel Analysis for the Alkaline Oxygen Evolution Reaction on NiFeOOH Electrocatalysts. *Angew. Chemie Int. Ed.* **2023**, *62* (7), e202216477. https://doi.org/10.1002/anie.202216477.

(2) Watzele, S.; Bandarenka, A. S. Quick Determination of Electroactive Surface Area of Some Oxide Electrode Materials. *Electroanalysis* **2016**, *28* (10), 2394–2399. https://doi.org/10.1002/elan.201600178.

(3) Wan, C.; Ling, Y.; Wang, S.; Pu, H.; Huang, Y.; Duan, X. Unraveling and Resolving the Inconsistencies in Tafel Analysis for Hydrogen Evolution Reactions. *ACS Cent. Sci.* **2024**. https://doi.org/10.1021/acscentsci.3c01439.
